# Formation of AAV Single Stranded DNA Genome from a Circular Plasmid in *Saccharomyces cerevisiae*

**DOI:** 10.1371/journal.pone.0023474

**Published:** 2011-08-10

**Authors:** Tiziana Cervelli, Ana Backovic, Alvaro Galli

**Affiliations:** 1 Laboratorio di Terapia Genica e Molecolare, Istituto di Fisiologia Clinica, CNR, Pisa, Italy; 2 Laboratorio di Biologia Molecolare, Scuola Normale Superiore, Pisa, Italy; Tulane University Health Sciences Center, United States of America

## Abstract

Adeno-associated virus (AAV)-based vectors are promising tools for targeted transfer in gene therapy studies. Many efforts have been accomplished to improve production and purification methods. We thought to develop a simple eukaryotic system allowing AAV replication which could provide an excellent opportunity for studying AAV biology and, more importantly, for AAV vector production. It has been shown that yeast *Saccharomyces cerevisiae* is able to replicate and form the capsid of many viruses. We investigated the ability of the yeast *Saccharomyces cerevisiae* to carry out the replication of a recombinant AAV (rAAV). When a plasmid containing a rAAV genome in which the cap gene was replaced with the *S. cerevisiae* URA3 gene, was co-transformed in yeast with a plasmid expressing Rep68, a significant number of URA3^+^ clones were scored (more than 30-fold over controls). Molecular analysis of low molecular weight DNA by Southern blotting revealed that single stranded DNA is formed and that the plasmid is entirely replicated. The ssDNA contains the ITRs, URA3 gene and also vector sequences suggesting the presence of two distinct molecules. Its formation was dependent on Rep68 expression and ITR. These data indicate that DNA is not obtained by the canonical AAV replication pathway.

## Introduction

Adeno-associated virus type 2 (AAV) is a non-pathogenic human *Parvovirus* of the *Dependovirus* genus that relies on a co-infecting helper virus such as *Adenovirus* or *Herpes simplex* virus for productive replication. In the absence of helper virus, AAV establishes a latent infection within the cell, either by site-specific integration into the host genome or by persisting in episomal forms. The 4.7 kb single-stranded (ss) DNA genome consists of two large open reading frames, named rep and cap, flanked by two 145 bp inverted terminal repeats (ITRs). The ITRs are the essential *cis*-acting elements required for genome replication and packaging. Cap encodes for the three structural proteins, VP1, VP2 and VP3 that form the viral capsid; rep encodes for four overlapping regulatory Rep proteins, Rep78, Rep68, Rep52 and Rep40 [1]. The two largest isoforms, Rep78 and Rep68, are necessary for AAV-2 replication [2] as well as for site-specific integration [3] and for transcriptional regulation of viral and cellular promoters [4]. Rep68 and 78 participate in the AAV DNA replication process via their interaction with Rep-binding element (RBE) and terminal resolution site (*trs*) sequences located within the ITRs [5], [6]. The Rep52 and Rep40 proteins are involved in the generation and accumulation of ssDNA viral genomes from double-stranded replicative intermediates [7], [8].

Over the last few years, viral vectors based on AAV have gained increasing popularity due to several favorable properties, including the high efficiency of transduction of post-mitotic tissues such as muscle, heart, brain and retina, and the long-term persistence of transgene expression in the absence of inflammatory or immune response [9], [10], [11]. Moreover, preclinical and early phase clinical trials in cystic fibrosis indicated that *in vivo* gene transfer by AAV-based viral vectors is feasible and relatively safe [12], [13]. Furthermore, AAV vectors are also able to target homologous chromosomal DNA sequences in mammalian cells at high frequency: precise, site-specific modifications were introduced in up to 1% of cells transduced with such vectors [14].

The principal limitation in the AAV vector usage for gene therapy is the production of the virus. The development of up-scalable, transfection-independent methods for rAAV production has been pursued, spurred by the requirement for large amounts of highly purified vector particles to perform experiments in large animal models and human clinical trials. Standard protocols for the generation of rAAV-based gene transfer vectors require co-transfection of cells with a therapeutic gene flanked by ITRs, and a packaging plasmid that provides the AAV Rep and Cap proteins. The transfected cells must also be over infected with a helper virus, e.g., *Adenovirus*, which can contaminate the rAAV stocks. To overcome this problem, a packaging/helper plasmid containing all AAV and *Adenovirus* functions required for amplification and packaging of AAV vector constructs has been generated [15]. Even if the production of helper virus-free rAAV stocks is obtainable, the titer of viral stocks is still highly dependent on transfection efficiency and on the particular human cell line utilized. An alternative method has been developed using the *Baculovirus* to provide the functions necessary for rAAV [16]. In view of the greater complexity of the cell biology and genetics of metazoans, we explored the possibility to obtain AAV genome replication in the yeast *Saccharomyces cerevisiae*. Thanks to the high evolutionary conservation of fundamental biochemical pathways, yeast has been and is currently used to clarify biological processes of multicellular eukaryotic organisms [17]. Yeast offers the advantage to be easily cultured and genetically manipulated. Moreover, this microorganism has already demonstrated its usefulness for virus research: many RNA or DNA viruses infecting plants (such as some members of the *Bromoviridae*, *Tombusviridae* and *Geminiviridae* and *Avsunviroidae* families), animals (such as the *Flock House*, *Nodamura* and *Bovine Papilloma viruses*) or humans (*Human Papilloma Virus*), replicate in yeast [18], [19], [20]. Furthermore, yeast has been utilized to produce vaccines for *Hepatitis B* [21] and for *Papilloma viruses* [22], for drug discovery [23] and to elucidate the function of individual proteins from important pathogenic viruses such as HIV, *Hepatitis C virus* and *Epstein−Barr virus* [18].

Accordingly, we designed an rAAV vector (pAAVRepURA3) containing the yeast marker URA3 between the ITRs in order to allow selection for yeast transformants in a URA3-deleted strain. The results reported here show that the ss DNA AAV genome is formed in yeast and suggest that this microorganism could be a promising tool to study in detail the role of host recombination, repair and replication proteins in AAV replication.

## Materials and Methods

### Plasmid construction

The plasmid pAAVRepURA3 is derived from pSub201 which contains the complete AAV-2 genome [24]. The pAAVRepURA3 vector maintains the entire rep gene and has an internal deletion of cap gene. The *URA3* marker gene, obtained from pMA150 digested with *Bam*HI [25], was cloned in the *Apa*I/*Kpn*I sites of pSub201 after filling in with Klenow polymerase. pAAVpokURA contains only the *URA3* gene and a stuffer sequence in order to increase the distance between ITRs. The stuffer sequence, denominated pok, consists of 2.5 kb of the mouse pokemon gene. pAAVpokURA was obtained cloning the pok sequence and URA3 in vector pMCSsub. pMCSsub was obtained by substituting the multiple cloning site of the plasmid pCMV-MCS (Stratagene, La Jolla, CA,USA) with a multiple cloning site containing *No*tI *Xba*I *Xho*I *EcoR*I *BamH*I *Hind*III *Sal*I *Xba*I *Not*I. The *URA3* gene was cloned in the *Hind*III site of pMCSsub, obtaining pMCSsubURA. The pok sequence was cloned in *Xho*I and *EcoR*I sites of pMCSsubURA obtaining pMCSubpokURA. Pok and URA3 were then cut out with *Xba*I and cloned in *Xba*I site of pSub201. pRepURA was obtained digesting the pAAVRepURA plasmid with *Xba*I to eliminate ITRs, and cloning the *Xba*I RepURA *Xba*I fragment in pMCSsub.

The episomal plasmids pGAD424, pG.Rep68 were kindly provided by M. D. Weitzman [26]. The pGAD424 contains the LEU2 marker. The pG.Rep68 plasmid contain respectively Rep68 and Rep40 genes under the control of constitutive yeast promoter ADH1 and the LEU2 marker.

### Yeast strain and transformation

The yeast strain RSY12 (*MATa leu2–3,112 his3–11,15 URA3::HIS3*) has a complete deletion of *URA3* gene which was replaced with the *HIS3* gene [27]. Complete (YPAD) and synthetic complete (SC) medium were prepared according to the standard procedures. The same amount of plasmid DNAs was co-transformed into yeast using the standard lithium chloride method with single-stranded DNA as carrier [28]. Transformants were selected onto SC-uracil (SC-URA) SC-leucine (SC-LEU) plates. The frequency of URA3^+^LEU2^+^ colonies was calculated dividing the number of URA3^+^LEU2^+^ colonies by the number of LEU2^+^ total transformants.

The yeast clone expressing Rep68 and Rep40 protein was obtained transforming the plasmid pG.Rep68 digested with *Sph*I to cut out 2 micron sequence in order to avoid autonomous replication of the plasmid [26].

### Total protein extraction and Western blotting

For analysis of protein expression by gel electrophoresis, total cell proteins were obtained by two rounds of protein extraction. 100–200×10^6^ of actively growing yeast cells were pelleted and subjected to the first round of extraction by the method published by Kushnirov [29]. Briefly, cells were pelleted and shortly exposed to mild alkali conditions (0.1 M NaOH) and lysed by heat at 95°C for 5 min in 50 µl of SDS-PAGE loading buffer 4X (150 mM TrisHCl pH 6.8, 50% glycerol, 10% SDS, 10% β-mercaptoethanol, 0.06% bromophenol blue). After eliminating cellular debris by centrifugation, the first protein extract named “first extraction” was ready for analysis by gel electrophoresis. For the “second extraction”, cellular debris was treated with a variant of a standard harsh RIPA buffer (500 mM NaCl, 50 mM Tris-HCl, 1 mM EDTA, 1% Triton, 1% DOC, 1% SDS) and put forward to a quick sonication (Soniprep 150). Precisely, the remaining pellet was resuspended in 140 µl of RIPA buffer (proteinase inhibitors added), incubated on ice for 20 min and then for another 20 min on the rotation at 4°C. After sonication (5 cycles of 10 seconds on and 20 seconds off; 15 microns amplitude), the second protein extract, named “second extraction”, was purified from remaining cellular impurities by quick centrifugation and analyzed by gel electrophoresis. Proteins were resolved on 10% polyacrylamide gel in SDS–Tris–glycine buffer. Following SDS-PAGE proteins were electro-transferred to Hybond-C Extra nitrocellulose membrane (Amersham Biosciences, UK) in methanol-Tris-glycine buffer. The blots were blocked with 10% milk in TBS-T (Tween 0.1%) for 1 h and then, incubated overnight at 4°C with the primary antibody anti-Rep monoclonal antibody 303.9 (Progen Biotechnik GmbH, Heidelberg, Germany) diluted 1∶50 in TBS-T with 5% milk. For the loading control the same blots were incubated for 1 h with primary mouse antibody anti-3 phosphoglycerate kinase (3PGK, ,Molecular Probes) diluited 1∶5000 in 1% milk in TBS-T. Secondary antibodies conjugated to horseradish peroxidase (IgG-HRP) (Santa Cruz Biotechnology, Inc.) were used for detection of specific antibody binding (diluted 1∶2500 in the same solution of the corresponding primary antibody). The blots were stained with the Super Signal West Femto chemiluminescent substrate (Thermo Fisher Scientific, Inc.).

### DNA isolation and Southern blotting

Genomic DNA was isolated using the Master pure yeast DNA purification Kit (Epicentre Biotechologies). Low molecular weight (M_r_) DNA was isolated from 5 to 10 independent URA3^+^LEU2^+^ clones harboring the AAV genome by using the yeast plasmid miniprep kit (Zymo Research Orange, CA) as previously reported [30]. The *Dpn*I resistance assay was performed to check that the plasmid had replicated in yeast. When the plasmid DNA is replicated and methylated by bacteria, it becomes sensitive to *Dpn*I digestion and it is thus distinguishable from DNA synthesized in eukaryotic cells which is not methylated and, therefore, resistant to digestion [31]. By contrast, *Mbo*I recognizes the same sequence as *Dpn*I, but cuts only DNA not methylated. The DNA samples were digested with *Dpn*I or *Mbo*I for 24 h and loaded on 1% agarose gel. The DNA was electrophoresed, transferred to nylon membrane (Roche) and probed with DIG-labelled URA3 obtained by PCR from the plasmid YEplac211 with following primers: pURA-R 5′GATACCCGGGACCTGACGTCTAAG3′ and pURA-F 5′ACATCCCGGGTGACGGGCTTGTCTGCTCCC3′. The ITR probe was obtained by DIG random primed labelling kit (Roche) using as template gel purified ITRs obtained digesting pSub201 with *Xba*I and *Pvu*II. To determine the presence of the plasmid backbone, we also constructed the F1 probe that was obtained by PCR performed with nucleotide mix containing DIG-dUTP from pSub201 plasmid with the following primers F1 forward: 5′CAA CCA TAG TAC GCG CCC 3′; F1 reverse: 5′ CAA TAA ATC ATA CAG GCA AGG 3′.

To determine the polarity of ssDNA we used two synthetic oligonucleotides of 100 mer as probe (IDT integrated DNA technologies). The probe for the plus (+) strand, named URA(+) has the following sequence: 5′CTTCCAACAATAATAATGTCAGATCCTGTAGAGACCACATCATCCACGGTTCTATACTGTTGACCCAATGCGTCTCCCTTGTCATCTAAACCCACACCGG 3′. To determine the presence of the minus (-) strand, we used the synthetic URA(-) probe with the following sequence: 5′CCGGTGTGGGTTTAGATGACAAGGGAGACGCATTGGGTCAACAGTATAGAACCGTGGATGATGTGGTCTCTACAGGATCTGACATTATTATTGTTGGAAG 3′. The probes were labelled using the DIG oligonucleotide 3′-End Labelling kit, 2nd generation (Roche), according to manufacturer's instructions. Equal amount of DNA was loaded on two gel slots, electrophoresed and transferred on nylon membrane positively charged (Roche). Since the 3′end labelled oligonucleotide probes are less sensitive than the PCR labelled probes, we hybridized only the ssDNA to increase the signal.

### S1 and Mung Bean reaction

To identify the presence of ssAAV genome, low M_r_ DNA samples were digested with S1 nuclease (Promega) or *Mung Bean* Nuclease (New England Biolabs) which cut only ssDNA. Briefly, 10 µl of low M_r_ DNA were digested with 0.1U of S1 nuclease for 30 min at 37°C in the provided buffer and inactivated at 70°C for 10 min. For *Mung Bean* Nuclease, 10 µl of low M_r_ DNA were digested with 0.1 U of *Mung Bean* for 30 min at 37°C in the provided buffer. The reaction was stopped with STOP solution (5 mM EDTA, 30 mM TrisHCl pH 7.5) followed by heating at 65°C for 10 min.

## Results

### Rep68 increases the frequency of yeast clones containing the AAV genome

**Figure 1:**
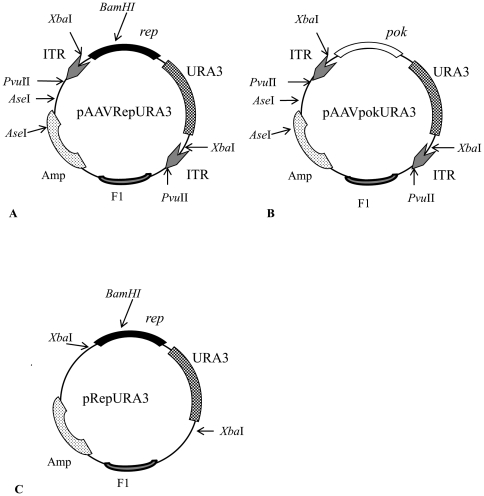
Schematic representation of plasmids. A) The vector pAAVRepURA contains wild type *Rep* gene and the yeast *URA3* marker replacing the *Cap* gene flanked by the two ITRs. B) The vector pAAVpokURA contains a stuffer sequence, pok, instead of *Rep* gene. The ITRs are the only AAV sequence present in the vector. C) The vector pRepURA has the same sequences as pAAVRepURA without ITRs.

To explore whether the yeast *Saccharomyces cerevisiae* is able to sustain the rescue of the AAV genome from a plasmid containing the ITRs, we constructed the pAAVRepURA3 vector containing the wild type rep gene and the *URA3* marker flanked by the ITRs (Fig.1A) and the pAAVpokURA vector which does not contain any AAV sequence, but only URA3 gene and the stuffer sequence, pok (Fig. 1B). With the vector pAAVpokURA we want to determine whether the internal Rep sequence may affect ssDNA formation. These vectors do not share any homology to the genome of the RSY12 strain which harbors the complete deletion of the chromosomal *URA3* gene [32]. Moreover, as pAAVRepURA3 and pAAVpokURA do not carry any yeast replication origin such as ARS or 2 micron, they cannot give rise to URA3 colonies unless they integrate in the yeast genome or replicate extra chromosomally.

**Figure 2:**
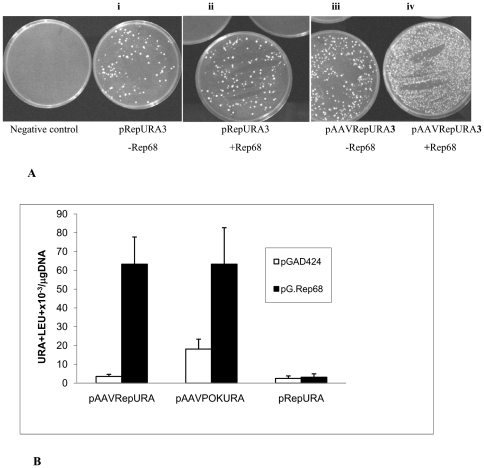
The frequency of colonies carrying the AAV genome increased when Rep68 is expressed. The plasmid pAAVRepURA3, pAAVpokURA linearized with *Pvu*II and the control plasmid, pRepURA3, digested with *Xba*I, were co-transformed with the plasmid pGAD424 or pG.Rep68 containing the *LEU2* marker gene (A, B). (A) Representative plates comparing colonies obtained from the transformed yeast RSY12 strain with plasmid pRepURA and pGAD424 (i), pRepURA and pG.Rep68 (ii), pAAVRepURA and pGAD424 (iii); pAAVRepURA and pG.Rep68 (iv). (B) Transformed yeast cells were scored for their ability to form colonies on selective medium lacking leucine and uracil. The frequency was calculated as described in material and methods. Results are the mean of 4 independent experiments±standard deviation.

The pAAVRepURA3 and pAAVpokURA were co-transformed into the strain RSY12 with the plasmid containing the Rep68 expression cassette, or with the control plasmid pGAD424. The plasmids pGAD424 and pGRep68 carry the LEU2 gene as yeast selection marker . As shown by counting the number of colonies grown onto the plates with medium lacking both uracil and leucine (Fig. 2A), the frequency of URA3^+^LEU2^+^ colonies increased from 3.05±1.1×10^−3^ (pAAVRepURA3 and pGAD424) to 91.7±43.6×10^−3^ (pAAVRepURA3 with pG.Rep68). Similarly, the frequency of URA3^+^LEU2^+^ colonies rose from 18.1±5.2×10^−3^ (pAAVpokURA with pGAD424) to 63.3±19.4×10^−3^ (pAAVpokURA with pG.Rep68) (Fig. 2B). The expression of Rep68 did not change the frequency of URA3^+^LEU2^+^ colonies when yeast was transformed with the plasmid pRepURA3 which does not contains the ITRs (fig 2B). These results suggest that the effect of the Rep68 is related to the presence of ITRs and that Rep68 may be important for AAV replication and rescue in yeast as in human cells.

### Rep protein expression in yeast

**Figure 3:**
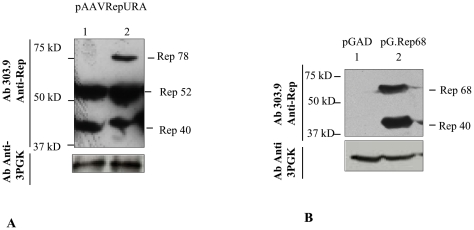
Rep protein expression in yeast. Western blotting of total yeast cell lysate from cells transformed with pAAVRepURA (A) and pG.Rep68 (B). (A) The two lanes were loaded with the protein obtained with first extraction (lane 1) and second extraction (lane 2). The second extraction contains Rep78, not present in the first extraction. (B) The two lanes were loaded with proteins from the second extraction of a yeast clone obtained from transformation with control plasmid pGAD (lane 1) and pG.Rep68 (lane 2). In (A) and (B), the amount of proteins loaded was detected with 3-phoshoglycerate kinase antibody (3-PGK).

We tested whether the natural AAV promoter p5 and p19 are able to drive the transcription of Rep open reading frame in yeast as it has been already demonstrated in human cells [7]. We performed western blot analysis of the total protein extracts from yeast cells transformed with the vector pAAVRepURA and grown in medium without uracil for several generations. As shown in the figure 3A, either in first extraction (lane 1) either in second extraction (lane 2) Rep40 and Rep52 are present, while Rep78 is observed only in second extraction (Fig 3A, lane 2). Rep68 is not observed. This observation suggests that natural AAV promoters are recognized by the yeast transcriptional machinery. The pG.Rep68 plasmid contains rep68 and rep40 genes under the control of constitutive yeast promoter ADH1 and the LEU2 marker [26]. Protein expression after transformation with this plasmid was analyzed by western blotting as shown in figure 3B (lane 2). Expression of both Rep68 and Rep40 was observed in the yeast strain carrying the pG.Rep68.

### AAV ssDNA formation in yeast

**Figure 4:**
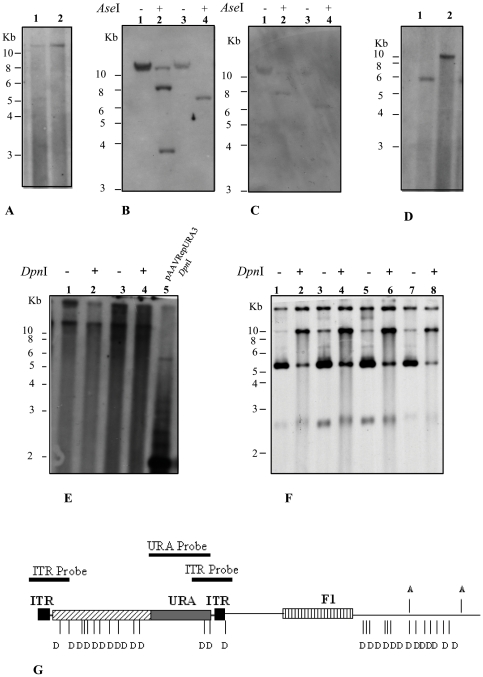
AAV replication in yeast. (A) Southern blot analysis of low M_r_ DNA of two different yeast clones URA3^+^ (lane 1 and 2) derived from transformation with pAAVRepURA3 using the URA3 marker gene as probe. (B, C) Southern blot analysis of genomic DNA of the same two clones as in B undigested (lane 1 and 3), and digested with *Ase*I (lane 2 and 4) probed with URA3 (B) or ITR probe (C). (D) Low M_r_ DNA of the two yeast clones URA3^+^LEU2^+^ derived from transformation with pAAVRepURA3 and successively transformed with pG.Rep68 (lane 1 and 2) analyzed on Southern Blot using the URA3 probe. (E) Low M_r_ DNA of yeast clones URA3^+^LEU2^+^ derived from co-transformation of pAAVRepURA with control plasmid pGAD424. Lanes 1 and 3 show the undigested DNA and lanes 2 and 4, DNA digested with *Dpn*I and subjected to Southern blot analysis using URA3 marker gene as probe. Lane 5 shows the result of *Dpn*I digestion of the pAAVRepURA plasmid. *Dpn*I was performed in order to demonstrate that AAV DNA replicated in yeast. (F) Low M_r_ DNA of yeast clones URA3^+^LEU2^+^ derived from co-transformation of pAAVRepURA with plasmid pG.Rep68. Lanes 1, 3, 5, 7 show the undigested DNA and lanes 2, 4, 6, 8 DNA digested with *Dpn*I and subjected to Southern blot analysis using URA3 marker gene as probe. (G) Schematic representation of *Dpn*I/*Mbo*I (indicated with D) and *Ase*I (indicated with A) restriction map of pAAVRepURA plasmid. The *Dpn*I/*Mbo*I restriction endonucleases do not cut in the URA3 gene.

The AAV genome inserted into a plasmid vector can initiate a productive AAV replication when it is transfected in human cells that are simultaneously or subsequently infected with a helper virus. The AAV genome is released from a circular plasmid in a way that is similar to the rescue of the integrated AAV provirus in latent phase [24]. It has also been observed that the rescue of the AAV genome in HeLa cells extracts is more efficient when the Rep68 protein is expressed [2]. We, therefore, checked if Rep proteins expressed from pAAVRepURA were sufficient to rescue AAV genome from the circular plasmid in yeast. To do so, low M_r_ DNA from URA3^+^ yeast clones transformed with the pAAVRepURA was analyzed by Southern blot and probed with *URA3* gene to check for the presence of rescued ssDNA that is expected to be about 3 kb (Fig. 4A). As we observed a band with a molecular weight higher than 10 kb, we conclude that rescue does not occur and that the URA^+^ phenotype is determined by integration of the vector in the genomic DNA. We therefore, analyzed the genomic DNA extracted from these clones after digestion with *Ase*I, that cuts the pAAVRepURA backbone in two sites but not the AAV sequence (Fig. 4G). The analysis revealed the presence of multiple (fig 4B and C, lane 2, or single integrated genomes (fig 4B and C, lane 4). This result suggests that a certain level of Rep proteins might be necessary to keep the pAAVRepURA plasmid replicating autonomously inside the yeast cell. The Rep protein expression can be directed by the AAV natural promoters p5 and p19 as already shown, but if Rep is required for AAV replication, we hypothesized that it has to be located in another vector in order to provide a certain amount of Rep to initiate the AAV genome replication. It is likely that the pAAVRepURA vector is maintained as an episome only when Rep proteins have reached a quite high level of expression. To verify this idea, we transformed yeast cells that have the pAAVRepURA integrated in the genome with the plasmid pG.Rep68. We extracted the low M_r_ DNA from two URA3^+^LEU2^+^ clones and performed Southern blot experiments. This analysis revealed the presence of only a band of ∼6 kb in one clone (Fig. 4D, lane 1) and a band with a molecular weight higher than 10 kb in the another clone (Fig. 4D, lane 2). The band of 6 kb could be due to a plasmid excision event occurring by intrachromosomal recombination that has been reported to occur at high frequency in haploid yeast strain [33], [34]. We, then, investigated the possibility that AAV ssDNA rescue took place after the co-transformation of pG.Rep68 and pAAVRepURA. Under these experimental conditions, yeast may have a the level of Rep proteins necessary to induce AAV ssDNA rescue from the transformed plasmid. Low M_r_ DNA was extracted from several independent clones, restricted with *Dpn*I and analyzed by Southern blot using the URA3 as probe. *Dpn*I digestion allows discriminating between DNA replicated in yeast from that replicated in bacteria because it cleaves only double-stranded sequences methylated or hemimethylated by *Dam* methylase. As shown in the figure 4E in two clones transformed with the pAAVRepURA and the empty pGAD, we mainly observed that the highest band is digested by *Dpn*I (Fig. 4E, lane 2 and 4). This suggests that the band corresponds to the transformed plasmid; the other band, higher than 10 kb, that is resistant to *Dpn*I digestion, corresponds to genomic DNA; in fact, probing low M_r_ DNA from the same clones with the genomic marker ADE2, the same band was observed (data not shown). In the two clones, no band corresponding to ssDNA was observed. When we analyzed low M_r_ DNA extracted from clones derived from yeast cells co-transformed with pAAVRepURA and pG.Rep68, we observed four main bands: a band higher than 10 kb that is genomic DNA, a band of about 10 kb, and two smaller bands of about 5.5 kb and 2.5–3 kb (Fig. 4F, lanes 1, 3, 5, 7). The *Dpn*I restriction did not change the band pattern, but determined a decrease in intensity of the 5.5 kb band and an increase in intensity of the 10 kb band consistent with a nicking activity of *Dpn*I (Fig. 4F, lanes 2, 4, 6, 8). As the plasmid pAAVRepURA contains 27 *Dpn*I sites (Fig. 4G) and some of them are really close to each other (less than 10 nucleotides apart), if one or more sites are nicked, it may result in a modification of the tertiary structure leading from a supercoiled circle (5.5 kb) to a nicked one (10 kb). Moreover, pAAVRepURA has a region of approximately 2 kb including the URA3 gene, which is free of *Dpn*I sites (Fig 4G). Thus, if *Dpn*I restriction had occurred, we would have observed the 2 kb band corresponding to URA3 gene as seen in the case of the plasmid pAAVRepURA extracted from bacteria (Fig.4E, lane 5). These results strongly suggest the presence of newly replicated episomal DNA in yeast clones.

The 2.5–3 kb band was not restricted by *Dpn*I suggesting that is a product of new replication and could be the ssDNA genome of AAV (Fig 4F, lanes 2, 4, 6, 8). The molecular weight corresponds to the ssDNA progeny of AAV observed by Wang *et al.* [35]

**Figure 5:**
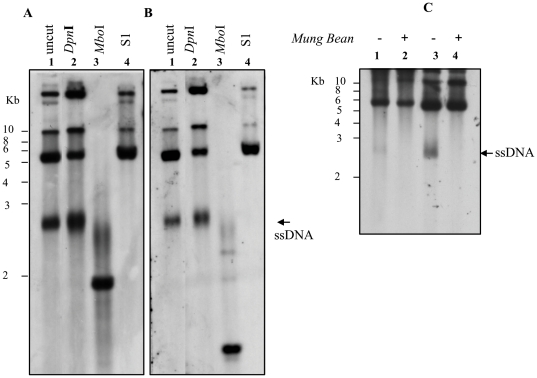
S1 and *Mung Bean* nuclease sensitivity. (A, B) Low M_r_ DNA from a URA3^+^LEU2^+^ clone derived from co-transformation of pAAVRepURA with plasmid pG.Rep68, was digested with *Dpn*I (loaded in the lane 2), *Mbo*I (loaded in the lane 3) , S1 nuclease (in the lane 4) and was analyzed on Southern Blot using URA3 probe (A) or ITR probe (B). (C) Southern blot analysis of low M_r_ of two URA3^+^LEU2^+^ clone derived from co-transformation of pAAVRepURA with plasmid pG.Rep68 was digested with *Mung Bean* nuclease (in the lane lane 2 and 4) and compared with the not digested DNA (lane 1, 3). DNA was detected using the URA3 probe. The arrows indicate the ssDNA which disappeared after digestion with S1 and *Mung Bean* nuclease.

A further demonstration that the three bands are the product of new replication of plasmid DNA is presented in figure 5A and B. Low M_r_ DNA extracted from one clone shown in Fig.4G is digested with the *Mbo*I restriction enzyme which recognizes the same palindromic sequence as *Dpn*I but cleaves only non-methylated DNA, such as the DNA replicated in yeast (Fig. 4A). The not digested DNA shows the same bands when probed with URA3 (Fig 5A, lane 1) or ITR (Fig 5B, lane 1) suggesting that contains the complete sequence of recombinant AAV. Digestion with *Mbo*I enzyme degraded completely the 10 kb and the 5.5 kb bands and produced the 2 kb band detected by URA3 probe (Fig.5A, lane 3) and not by ITR probe (Fig.5B, lane 3), suggesting that they are mainly newly replicated DNA. The 2.5–3 kb molecule is only partially digested by *Mbo*I and not completely degraded as the other bands. This observation does not rule out the possibility that the band represents the ssDNA because it has been demonstrated that some restriction enzymes, including *Mbo*I, are able to cut ssDNA [36], [37].

### Characterization of ssDNA

In order to ascertain that the lowest band corresponds to ssDNA AAV genome, we exposed the extracted DNA to the single-stranded DNA-specific S1 or *Mung Bean* nuclease. Low M_r_ DNA digested with the S1 or *Mung Bean* nuclease was analyzed by Southern blot and probed with URA3 or ITRs. As shown in figure 5, in the lanes where the DNA samples treated with the S1 nuclease were loaded (Fig. 5A, lane 4; fig 5B, lane 4) the 2.5–3 kb band is not present. The same result was observed when the DNA was treated with *Mung Bean* nuclease (Fig. 5C, lanes 2 and 4). Importantly, when the probe ITRs was used in the Southern blot (Fig. 5B), the three bands were again obtained, demonstrating that these replication-essential DNA elements are present.

### Characterization of replicated rAAV

**Figure 6:**
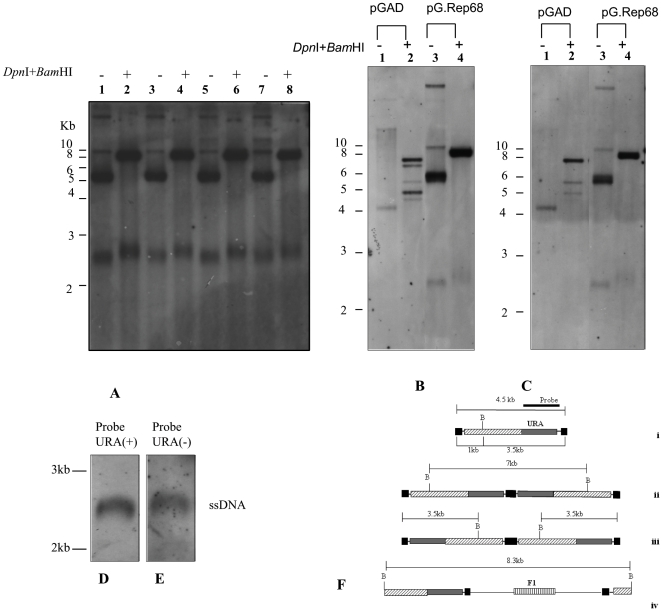
Characterization of newly replicated DNA. (A) Low M_r_ DNA of four clones derived from yeast co-transformed with pAAVRepURA and pG.Rep68 digested with *Dpn*I and *Bam*HI (lane 2, 4, 6, 8) and not digested (lane 1, 3, 5, 7) was analyzed on Southern Blot and probe with the URA3 gene. *Bam*HI cuts once in AAV construct. (B, C) Low M_r_ DNA of one clone obtained from the co-transformation of pAAVRepURA with pGAD not digested (lane 1) and digested with *Dpn*I and *Bam*HI (lane 2); the low M_r_ DNA of one out four clones analyzed in panel A undigested (lane 3) and digested with *Dpn*I and *Bam*HI (lane 4) was analyzed on Southern Blot and detected with URA3 probe (B) and F1 probe (C). (D, E) Equal amount of low M_r_ DNA of one out of four clones analyzed in panel A was loaded in two gel slots and transferred on nylon membrane. The membrane was cut in two pieces corresponding to gel slots and further cut to leave only ssDNA. The ssDNA was detected using as probe a 100-mer oligonucleotide complementary to the filament (+) of the URA gene URA(+) (D), or a oligonucleotide probe complementary to filament (-) of the URA gene URA(-) (E), to determine the polarity of ssDNA. (F) Restriction maps of predicted replicative intermediates: i) linear monomer, ii) tail-to-tail dimer, iii) head-to-head dimer, iv) linear vector. Position of *Bam*HI is indicated with B. The sizes of the fragments liberated following *Bam*HI cleavage and recognized by the URA probe are shown next to corresponding structures. ITRs are indicated as black boxes.

During the rescue of AAV from plasmid in 293T cells, it is possible to isolate the linear double-length double-stranded DNA molecules and the DNA double-stranded single copy [35]. According to the AAV DNA replication model, the secondary structure formed by the ITRs provides a free 3′ hydroxyl group for the initiation of viral DNA replication via a self-priming strand-displacement mechanism involving leading-strand synthesis and double-stranded replicative intermediates monomer formation (Fig.6F,i). These structure is resolved at the *trs* by site-specific nicking of the parental strand opposite the original 3′ end position (i.e., at nucleotide 125) leading to the formation of ssDNA. When nicking does not occur, elongation proceeds through the covalently closed hairpin structure generating linear double-length double-stranded molecules with either a tail-to-tail configuration (Fig.6F,ii) or a head-to-head configuration (Fig.6F,iii) [1]. To determine the structure of molecules replicated in yeast, we digested low M_r_ DNA with *Bam*HI that cuts pAAVRepURA only once in rep sequence at 1 kb (Fig 6F, i). If the newly replicated molecules would be generated according to AAV replication model observed in human cells, the *Bam*HI digestion of low M_r_ DNA detected with URA3 probe, would produce a band of 3.5 kb in case of linear monomer (Fig.6F,i); a band of 7 kb in case of tail to tail dimer (Fig.6F,ii); a band of 3.5 kb in case of head to head (Fig.6F,iii). As shown in Fig.6A, no one of this molecules was produced, but rather we found only one band of ∼8 kb (lanes 2, 4, 6, 8) which is the size of the linearized plasmid containing the backbone and AAV genome (Fig 6F,iv).

To confirm this hypothesis, we digested with *Bam*HI the low M_r_ DNA isolated from a URA3^+^LEU2^+^ clone carrying pAAVRepURA plus pGAD (Fig. 6B, lane 2; fig. 6C,lane 2) or pAAVRepURA plus pG.Rep68 (Fig.6B, lane 4; fig.6C,lane 4) and hybridised with the F1 (Fig.6B) that recognizes F1 origin of replication that is located in the backbone of the plasmid or URA3 (Fig.6C) that recognized the URA3 sequence in the AAv vector. As shown in figure 6B and 6C, the bands obtained with F1 probe are coincident with those obtained with URA3 probe. The results of the *Bam*HI digestion produce a band of ∼8 kb recognized by URA3 and F1 (Fig 6B lane 4; Fig 6C, lane 4). This observation indicates that the band of 5.5 kb corresponds to the supercoiled pAAVRepURA and the band of 10 kb corresponds to the nicked circular form. Moreover, the ssDNA molecules band is recognized by both probes indicating the presence of two different molecules, one containing AAV sequences and one with vector sequences. To determine the polarity of ssDNA, we detected the ssDNA with two oligonucleotides probes complementary to filament +, URA(+) (Fig. 6D) or to filament - URA(-) (Fig. 6E). As shown in figure 6D and E both the plus and minus strands of ssDNA are generated.

### ssDNA formation is independent of AAV Rep sequence and dependent on ITRs

**Figure 7:**
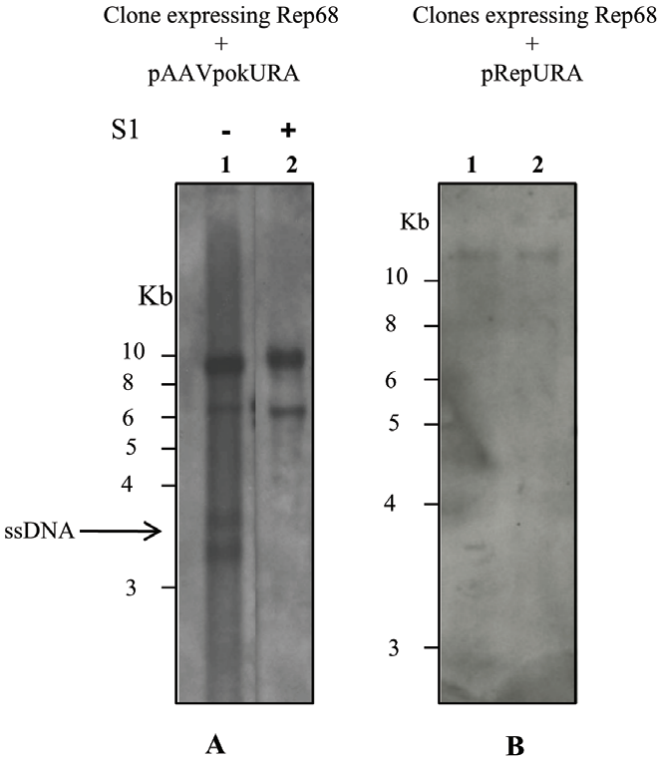
ssDNA formation is dependent on ITRs and Rep68. Southern blot of low M_r_ DNA obtained from a clone expressing Rep68 transformed with pAAVpokURA (A) or pRepURA (B) and probed with URA3. (A) Lane 1 shows low M_r_ DNA undigested and lane 2, low M_r_ DNA subjected to S1 nuclease. (B) Low M_r_ DNA of two different clones (lane 1 and 2) expressing Rep68 and transformed with pRepURA.

Recombinant AAV vectors do not contain AAV genome sequences but only the transgene that can be used for gene therapy in human cells. It has been shown that a region of the adeno-associated virus type 2 (AAV-2) *rep* gene acts as a *cis*-acting Rep-dependent element able to promote the replication of transiently transfected plasmids [38], [39]. We wondered if the formation of ssDNA was dependent on the presence of rep sequence in the vector. To address this point we constructed the pAAVpokURA that has *URA3* gene and a stuffer sequence denominated *pok* flanked by the two ITRs (Fig. 1B). In total the sequence corresponding to the AAV is 4.1 kb. This vector was transformed in yeast clone expressing Rep68 from an integrated copy of pG.Rep68 plasmid. Low M_r_ DNA was isolated, treated with S1 nuclease to ascertain the presence of ssDNA and analyzed by Southern blot. As shown in the figure 7A lane1, the ssDNA is produced. The two bands corresponding to ssDNA observed could represent two different secondary structures of the molecule. This suggests that the formation of ssDNA is not dependent on rep binding element present in rep gene, but only on ITRs.

To verify if the ITRs are involved in the formation of the new replicated molecules and the ssDNA, we constructed the vector pRepURA. This vector contains the same sequences as pAAVRepURA but not the ITRs (Fig. 1C). Low M_r_ DNA isolated from two URA3^+^LEU2^+^ clones obtained transforming pRepURA in the yeast clone expressing Rep68 (Fig.7B, lanes 1, 2) was analyzed by Southern blot. As shown in figure 7B, neither circular DNA molecule nor ssDNA was generated in the presence of Rep68.

## Discussion

The results reported here establish the novel finding that the AAV ssDNA is formed in yeast from a double stranded circular plasmid containing the ITRs. Moreover, the plasmid is maintained through the generation as a double stranded circle in the absence of the 2 micron replication origin or ARS sequence. First, we simply constructed a AAV vector containing the wild type rep sequence and the URA3 marker between the two ITRs, in order to select yeast clones carrying the rAAV. Interestingly, we demonstrated that Rep protein expression occurs in yeast starting from the AAV promoter p5 and p19 (Fig. 3A). The failure to obtain the ssDNA genome when yeast was transformed only with pAAVRepURA vector it is likely due to the absence of Rep protein at the moment of transformation; in fact, when Rep protein was expressed from pG.Rep68 plasmid transformed together with a high amount of AAV vector, the frequency of URA3^+^LEU2^+^ colonies was increased. The transforming DNA carrying the rAAV could follow different ways: it could be recognized by the DNA nuclease and degraded, or be channeled in the recombination process and consequently integrated in the host cell genome. The transforming rAAV plasmid could also be replicated and the AAV genome be rescued from the plasmid. The latter process can occur only in the presence of Rep68 [2]. To test the hypothesis that ssDNA may be generated by plasmid replication, we co-transformed the rAAV vector with pG.Rep68, allowing the expression of Rep68 and Rep40 (Fig. 3B) in order to have high concentration of rAAV together with Rep proteins. In these conditions, we obtained the ssDNA as shown in figure 4F. Moreover, we also demonstrated that the ssDNA is formed only when the ITRs are present (Fig. 7B, lane 1 and 2).

**Figure 8:**
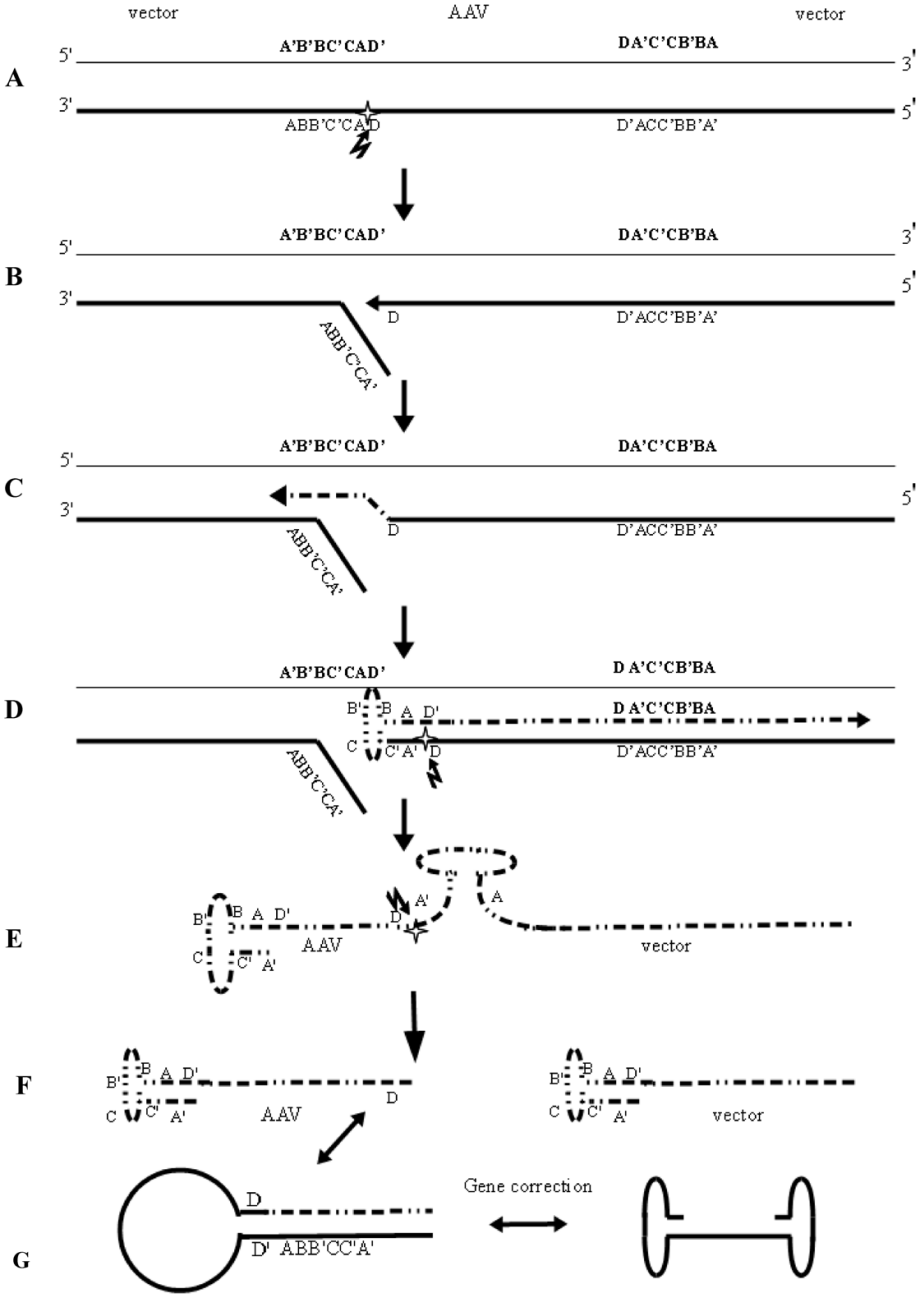
Diagram model showing ssDNA formation from a plasmid containing the AAV genome in yeast. (A) For the sake of clarity, the plasmid carrying ITRs is depicted as linear molecule. Rep nicks at the *trs* (white star) (B). A replication complex is assembled and replication commences through the ITR towards the vector (C). The new synthesized ITR fold into a hairpin conformation displacing the replication strand. Such displacement determines a template switch so that the originally nicked strand is copied during replication. The replication passes through the other ITR and proceeds into the vector sequence (D). After replication fork has completed a full circle, Rep produces a second nick and the newly synthesized DNA is displaced as ssDNA (E). The new ssDNA is nicked again and two ssDNA containing only one complete ITR are formed (F). The missing ITR is repaired by gene correction mechanism (G).

By using both *in vivo* and *in vitro* experimental systems, the principal features of the conventional AAV rescue model have been shown to include the synthesis of duplex linear replicative forms (monomers and dimers) that are self priming by virtue of terminal hairpin palindromes [35], [40], [41]. Two sort of mechanisms have been proposed to explain rescue of AAV in human cells: rescue may be carried out by repair cellular nucleases [42], [43], [44] or it may be coupled to DNA replication [2], [44], [45]. It has been observed that rescue of the AAV genome from a plasmid may be carried out by a Holliday structure-resolving activity *in vitro* [46] and i*n vivo* [47]. In any case, the episomal DNA is not produced by the “AAV rolling hairpin” type of DNA replication because ,when we analyzed the structure of low M_r_ DNA molecules (Fig. 6) we did not observe the canonical AAV replicative intermediates, but rather the supercoiled, nicked circular replicated plasmid and the ssDNA. Moreover, these molecules contain not only ITRs and URA3 sequences, but also vector sequences. This observation suggests that the entire plasmid is replicated as circle from which ssDNA is released. To explain the production of ssDNA genomes without generating linear duplex intermediate, we propose a model outlined in figure 8. According to our model, Rep68 binds the ITR at the RBE element and nicks one strand at the level of *trs* (Fig. 8A). The nick creates a free 3′-OH end from which DNA replication is initiated (Fig. 8B) [8]. As the replication passes through the body of ITR (Fig. 8C), the partially single stranded template strand will fold into a hairpin configuration (Fig. 8D). This folding of the ITR template may be supported by Rep binding to the tip of the hairpin loop of the ITR [43].This event produces a strand switch and the template is likely to be the nicked strand and not the complementary one. The replication fork proceeds through the vector sequence and terminate when reaches the nicked ITR. A second nick by Rep protein occurs at the level of the newly synthesized ssDNA that is, subsequently, displaced by the helicase activity of Rep proteins (Fig. 8D). The ssDNA molecule containing the entire plasmid sequence is nicked by Rep protein (Fig. 8E). The final products are two ssDNA molecules containing only one ITR; one with vector sequences and one with rep and URA3 genes (Fig. 8F). Our model predicts that the resulting ssDNA genome with AAV sequences has a complete ITR only at the 5′ end while the 3′ end carries only a D element. The missing ITR, however, can be repaired via a gene correction mechanism described by Samulski et al. [44]. This event involves the formation of a single stranded panhandle DNA intermediate in which both D elements are inverted and therefore can anneal to initiate the ITR repair synthesis (Fig. 8G) [48]. One ssDNA molecule has a complete ITR only at the 5′ end while the other one carries ITR sequences without D element. If the ITRs do not fold into a hairpin conformation, the replication continues on the backbone vector sequences and the plasmid is completely replicated as observed *in vitro* [2], [49]. In this case the ITR is used as a cellular origin of DNA replication [49]. We also validated our model by determining the polarity of the ssDNA. By using specific ssDNA probes, we demonstrated the both plus and minus AAV strands are formed in yeast (Fig. 6D, E). Moreover, this study demonstrated that in yeast the ITRs represent the only sequences required for ssDNA AAV formation from an episomal plasmid (Fig 7A, B).

Why does the yeast produce such high level of ssDNA? Is that due to an accumulation process of the newly formed ssDNA or is the mechanism leading to ssDNA formation more efficient in *Saccharomyces cerevisiae* than in human cells? The answer to these questions is not very easy but understanding the cellular factors involved in the ssDNA formation may be crucial for AAV encapsidation. It is known that yeast is very efficient in DNA repair mediated by homologous recombination. Yalkinoglu et al. [50] have shown that a plasmid containing the extreme left half of AAV with a single ITR could replicate in cells treated with genotoxic reagents suggesting a possible role of DNA damage repair in this process. Moreover, in cultured human cells, a marked increase in rAAV transduction efficiency is obtained by treating cells with agents that affect genomic DNA integrity or metabolism, such as UV irradiation, hydroxyurea (HU), topoisomerase inhibitors and several chemical carcinogens [51], [52], [53], [54]. It has been demonstrated recently that MRE11 complex, which is primarily involved in DNA double strand break repair, binds the incoming ssAAV genome and poses a barrier to AAV and that the helper function provided by the adenoviral proteins E1b55K/E4orf6 involves the degradation of MRE11 complex [55], [56]. These observations suggest that in human cells MRN complex can have a role in the rescue of AAV from plasmid. The higher efficiency of the non-homologous end-joining apparatus in mammalian cells, related to the strong pressure to repair double-strand breaks in these large genomes, probably explains the ability of AAV to grow in these cells only when that apparatus is partially saturated by double-strand break events. This requirement is apparently not present in yeast that yet seemingly contains all the cellular factors necessary for AAV genome replication in the right balance. In yeast, double strand breaks are mainly repaired by homologous recombination so the MRN complex proteins are available for other processes and could be immediately engaged in ssDNA formation. The formation of ssDNA does not occur immediately after pAAV plasmid transformation and required the replication of the plasmid. In fact, we did not detect any ssDNA in yeast cells 3 or 24 hours after transformation (data not shown). Therefore, we can speculate that the formation of ssDNA initiates after the plasmid has been replicated. It could be interesting to study AAV ssDNA genome in yeast cells with different genetic background. Thus, the utilization of this organism could allow not only to study the cellular requirements for viral production, but, not less importantly, to overcome the present limitations to produce rAAV for targeted gene therapy, since it is safe, cheap and amenable to easy manipulate the host.

We can thus conclude that the AAV genome can replicate in *Saccharomyces cerevisiae*; this is not surprising because yeast may replicate DNA genomes for other such as *Human papilloma virus* [57] and the ssDNA genome, *Indian Mung Bean Yellow Mosaic virus* [58].
